# Supplementary material for: Altered microRNA Transcriptome in Cultured Human Liver Cells upon Infection with Ebola Virus
Source: Int J Mol Sci. 2021 Apr 6;22(7):3792. doi: 10.3390/ijms22073792 (PMC8038836; doi:10.3390/ijms22073792)
Supplement: Supplementary file 1 [file ijms-22-03792-s001.zip › Supplementary_File/C_ GO_Analysis_Results/16-30nt_go_Makona-96h-Huh7_vs_Control-96h-Huh7_up.mature_mirna_targets/GeneOntology_FoldEnrichment.pdf]

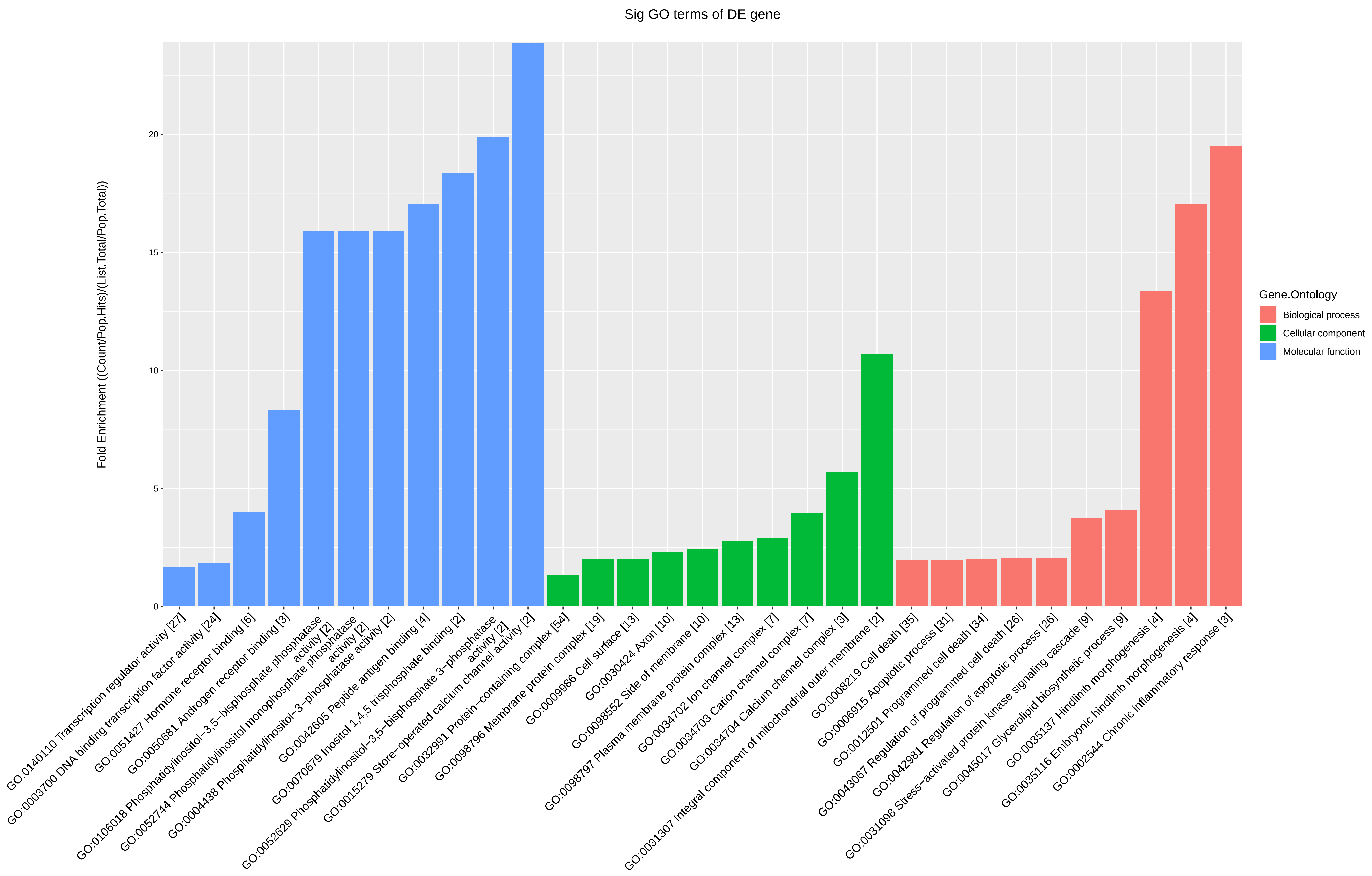
Sig GO terms of DE gene
Fold Enrichment ((Count/Pop.Hits)/(List.Total/Pop.Total))
0
5
10
15
20
GO:0140110 Transcription regulator activity [27]
GO:0003700 DNA binding transcription factor activity [24]
GO:0051427 Hormone receptor binding [6]
GO:0050681 Androgen receptor binding [3]
GO:0106018 Phosphatidylinositol−3,5−bisphosphate phosphatase activity [2]
GO:0052744 Phosphatidylinositol monophosphate phosphatase activity [2]
GO:0004438 Phosphatidylinositol−3−phosphatase activity [2]
GO:0042605 Peptide antigen binding [4]
GO:0070679 Inositol 1,4,5 trisphosphate binding [2]
GO:0052629 Phosphatidylinositol−3,5−bisphosphate 3−phosphatase activity [2]
GO:0015279 Store−operated calcium channel activity [2]
GO:0032991 Protein−containing complex [54]
GO:0098796 Membrane protein complex [19]
GO:0009986 Cell surface [13]
GO:0030424 Axon [10]
GO:0098552 Side of membrane [10]
GO:0098797 Plasma membrane protein complex [13]
GO:0034702 Ion channel complex [7]
GO:0034703 Cation channel complex [7]
GO:0034704 Calcium channel complex [3]
GO:0031307 Integral component of mitochondrial outer membrane [2]
GO:0008219 Cell death [35]
GO:0006915 Apoptotic process [31]
GO:0012501 Programmed cell death [34]
GO:0043067 Regulation of programmed cell death [26]
GO:0042981 Regulation of apoptotic process [26]
GO:0031098 Stress−activated protein kinase signaling cascade [9]
GO:0045017 Glycerolipid biosynthetic process [9]
GO:0035137 Hindlimb morphogenesis [4]
GO:0035116 Embryonic hindlimb morphogenesis [4]
GO:0002544 Chronic inflammatory response [3]
Gene.Ontology
Biological process
Cellular component
Molecular function
